# Optimization of magnetic coupling mechanism of dynamic wireless power transfer based on NSGA-II algorithm

**DOI:** 10.1038/s41598-024-55512-9

**Published:** 2024-03-01

**Authors:** Weihang Tang, Long Jing, Wanyu Cao, Wenzheng Xu, Xuezhi Wu, Hongbin Liao

**Affiliations:** 1 Beijing Jiaotong University, Beijing, China; 2 China Southern Power Grid, Guangzhou, China

**Keywords:** Engineering, Electrical and electronic engineering

## Abstract

Optimization of magnetic coupling mechanism is an important way to improve the performance of a dynamic wireless power transfer system. Inspired by the common radial magnetic core for circular coils, a new radial magnetic core for rectangular coils is adopt. Through simulation and experimental results comparison, which has higher coupling coefficient with the same core area. Combined with the magnetic circuit analysis, the magnetic flux leakage and conduction regions are divided into magnetic fluxes with different shapes, which magnetic resistances are calculated respectively. Based on the simulation results, parameter distributions of fluxes under different conditions are obtained. Therefore, the expressions of the coupling coefficient *k* of the adopt magnetic cores and coils and the design parameters of coils and cores are obtained. Taking the maximum *k* and the minimum rate of change of coupling coefficient with 100 mm displacement as the optimization objectives, a multi-objective optimization solution is carried out by using NSGA-II algorithm. The coil optimization scheme is obtained and verified by experiments. *k* and *Δk* are 0.442 and 6.8% respectively, and the errors are less than 5%. In the optimization process, there is no simulation model constructed. The optimization modeling combined of magnetic field segmentation method and parameter fitting has lower complexity and calculation time of optimization.

## Introduction

Wireless Power Transfer (WPT) technology is developing rapidly with the popularization of Electric Vehicle (EV) and Automatic Guided Vehicle (AGV). WPT technology can be divided into Static Wireless Power Transfer (SWPT) and Dynamic Wireless Power Transfer (DWPT) according to whether the vehicle is parking or driving during charging^1–5^. DWPT technology has attracted extensive attention in recent years because of non-contact power supply, higher safety, charging while driving and reducing charging anxiety^6,7^. In the field of EV and AGV wireless charging, DWPT technology usually transmits electric energy from the transmitter to the receiver through the coils at a transmission distance at cm level. The transmission power can be of the kilowatt level when the receiving coil moving^8^, and the transmission efficiency increases with the increase of the coupling coefficient of the transmitting and receiving coils^9,10^. Therefore, it is a significant way to optimize the shape, area, turns and cores distribution of the coupling mechanism to improve the coupling coefficient is a significant way for the enhancement of the transmission performance of the WPT system^11^.

Aiming at maximizing the coupling coefficient, several finite element simulation models were established, and the optimal design schemes of the coil were obtained through the simulation results under different coil and core parameters^12,13^. Resultantly, two new designs of core shapes were proposed^14,15^. Compared with the traditional core structure, it was verified that core shapes can reduce the eddy current loss of the magnetic sheet and improve the coupling effect. By the co-simulating based on MATLAB and Maxwell with the optimization algorithm, the coils and cores were optimized through parametric modeling to improve the coupling coefficient of the coil^16,17^. The above studies adopt a trial-and-error method based on the comparison of multiple simulation experiments, which takes a long time. With the change of the conditions, numerous models need to be constructed again.

The Fe-based nanocrystalline and nanocrystalline flake ribbon were used as shielding materials, which proved that the novel materials are more suitable for WPT application compared to traditional materials^18,19^. A model was built for the optimization of circular wireless charging coils for electric vehicles, which had optimization variables conclude the coil inner diameter, the number of turns and the core spacing^20^. Based on the second-generation non-dominated genetic algorithm (Non-dominated Sorting Genetic Algorithm II, NSGA-II), a multi-objective optimization of coupling coefficient and quality factor was performed. And the optimal solution achieved 1 kW power level^21^. With the center turn spacing, edge turn spacing and core width taken as optimization variables, and with the coupling coefficient and system cost taken as the optimization goals, the Optimization model of the Double-D coils and the magnetic cores was constructed, based on the two-dimensional finite element simulation results^22^. Based on the value of the optimization variables in each iteration, the next iteration can obtain the optimization scheme with the improving topology search algorithm^23^. The optimal distribution of single-column transmitting coils is determined by the calculation in the distributed wireless charging scheme, which can effectively solve the problem of severe fluctuation of output power in the case of displacement of the receiving coil in centralized wireless charging. However, the optimization of the core is not involved in the research^24^. Aiming at maximizing the output of the receiving coils, an optimization model was constructed to characterize the relationship between the output power of the distributed wireless charging system and the mutual inductance of coils. The optimal solution was obtained by an iterative optimization method^25^. Reference^26^, which is based on a 2D finite element simulation of a hybrid-wound non-contact transformer, established a magnetic circuit distribution equation, aiming at obtaining the maximum coupling coefficient and the minimum volume weight. By comparing the effects of the parameters in the coupling coefficient curve, the optimization scheme was obtained. The above studies analyzed the coil model, established the analytical expression, and optimized the solution through the optimization algorithms. However, though the core design schemes were obtained via the coil focused optimizations, the optimizations were only based on the simulations and the comparisons. Meanwhile, the cores and the coils were not analyzed as a whole. Moreover, to obtain the optimization result it still requires a large number of magnetic core simulation models, which prolongs the optimization process.

Compared with circular coil, rectangular planar coil, as a commonly-used type of coupling coils in DWPT system, can better utilize the space. When the length of the rectangular coil is the same as the diameter of the circular coil, the rectangular coil can occupy a larger area. And it is usually wound as fillet in actual winding so as to obtain higher coupling coefficient^27^. Compared with Double-D coils, the transmitter and receiver structure, which composed of a raw of rectangular coils and a single rectangular coil, has higher magnetic flux density and can manifest better coupling effect^28^. Therefore, this study focuses on the rectangular coil in the EV DWPT system, and discusses a radial magnetic core shape. It is verified that the core shape has better coupling effect than the common shapes. Based on the magnetic circuit analysis of rectangular coil, the expression between the coupling coefficient *k* and magnetic reluctance *R* is deduced of the whole structure with new type core and coil. The change of coupling coefficient is analyzed when the receiving coil is misaligned. Moreover, a multi-objective optimization model for dynamic wireless charging of electric vehicle is built, which takes the maximum coupling coefficient and the minimum sensitivity of coupling coefficient with displacement as the optimization objectives, whereas the length of the transmitting and receiving coil, the number of turns and the core width are taken as the optimization variables. The optimization solution of coils and cores is obtained by NSGA-II algorithm. The accuracy of the conclusion is verified by simulation and experiment.

This paper is structured as follows. In Section "DWPT system analysis", taking the S–S topology as an example, the influence of coupling coefficient on efficiency of the wireless charging system is discussed. In Section "Calculation of coupling coefficient based on magnetic circuit analysis", the calculation expression of the coupling coefficient based on magnetic circuit analysis is obtained. In Section "Coil optimization based on NSGA-II algorithm", the optimization modeling is optimized with genetic algorithm, which avoids the optimization process with multiple finite element simulation and comparison, shortens the optimization process. In Section "Experiment and Verification", three actual windings and the finite element simulation models with the same parameters of the optimization schemes are built and compared with the calculation solution, which proves the accuracy of the calculation expression and verifies the optimization results.

## DWPT system analysis

**Figure 1:**
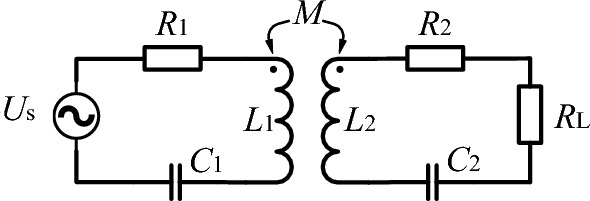
S–S equivalent circuit model.

In the design of the resonant circuit in the WPT system, according to the connection method of the coils and the capacitors, there are four fundamental compensation topologies: S–S, S–P, P–P, and P–S. In general, the inductor of coils and the capacitor should be in a resonant state to achieve the best transmission power and efficiency^29^. Taking the S–S structure as an example, the equivalent circuit model is shown in Fig. 1, where: *U*_s_ is the high-frequency power source, *R*_1_ and *R*_2_ are the resistances of the transmitting and receiving coils, *L*_1_ and *L*_2_ are the inductances of the transmitting and receiving coils, and *C*_1_, *C*_2_ are the transmitting and receiving side compensation capacitors, *M* is the mutual inductance, and *R*_L_ is the equivalent load.

The receiving side reflection impedance is

1 $$Z_{{{\text{re}}}} = \frac{{\omega^{2} M^{2} }}{{R_{2} + R_{L} }}$$

It can be seen from (1) that when the receiving side is in the resonance state, the reflected impedance is resistive. Therefore, the selection of the transmitting side compensation capacitor is only related to the selection of the transmitting side inductance of the coil, but is not affected by the receiving side parameters. The studies have shown that the S–S structure can be equivalent to a voltage source externally. Therefore, it is easy to achieve a large transmission power with a small coupling coefficient^30^, which is suitable for WPT of EV. So, this paper chooses S–S structure as a research object for wireless charging of electric vehicles.

The efficiency of the resonant circuit is

2 $$\eta = \frac{{P_{out} }}{{P_{in} }} = \frac{{R_{L} }}{{\frac{{(R_{2} + R_{L} )^{2} + R_{2} + R_{L} }}{{k^{2} Q_{1} Q_{2} R_{2} }}}}{ = }\frac{{k^{2} Q_{1} Q_{2} R_{2} R_{L} }}{{(R_{2} + R_{L} )(R_{2} + R_{L} + 1)}}$$

where *k* is the coupling coefficient, *Q*_1_ and *Q*_2_ are the quality factors of the transmitting and receiving coils respectively.

It can be seen from (2) that the efficiency of the DWPT system is positively correlated with the coupling coefficient *k*, so that *k* is a very important factor for increasing the efficiency of WPT systems^31^. So, the coil coupling coefficient *k* is taken as an optimization objective in this paper.

## Calculation of coupling coefficient based on magnetic circuit analysis

### A new radial core of rectangular coil

**Figure 2:**
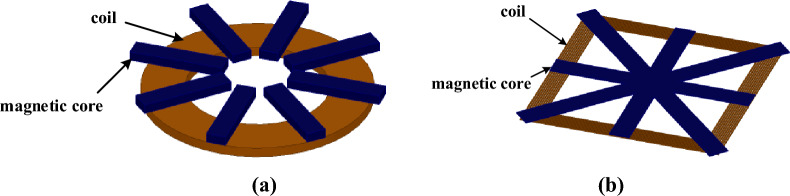
Radial cores structure. (**a**) a radial core for circular coil. (**b**)a new radial core for rectangular coil.

The parameters of the cores in the coupling mechanism include the number, length, width, thickness, and distribution of magnetic strip. A core structure of circular coil commonly used in the WPT system of EV is a radial, as shown in Fig. 2a^32,33^. Inspired by the radial core of circular coil, in order to improve the system coupling ability under the same area of the chassis of EV, this paper discusses a new core structure for rectangular coil. As shown in Fig. 2b, the coil is tightly wound around the magnetic core, which is composed of four magnetic transverse strips in different directions. The magnetic strips of the radial magnetic core are arranged as evenly distributed along the central line of the length, width direction and diagonal direction. The edges of magnetic strips are usually parallel to the coil edge or slightly beyond the coil edge.

As to the functions of the specific components of the oblique core strips can reduce the diagonal magnetic resistance within the coils and decrease the length of equivalent magnetic circuit and magnetic resistance. The protruding part of the magnetic strip outside the coil can enhance magnetic conduction and magnetic shielding. The radial core can enhance magnetic conduction in length and width directions just the same as the intersectional shaped core commonly used in rectangle coil. The magnetic stripes are evenly arranged along the length and width direction.

**Figure 3:**
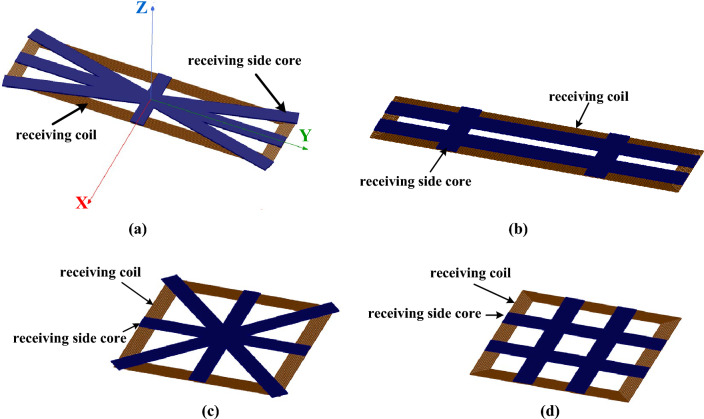
Simulation models in Maxwell. (**a**) 50 × 200cm radial core. (**b**) 50 × 200cm intersectional shaped core. (**c**) 100 × 100cm radial core. (**d**) 100 × 100cm intersectional shaped core.

In the finite element simulation software ANSYS MAXWELL 18.1, the models of intersectional shaped core and radial core are constructed respectively. The two shapes of the core, which is made of PC95 as material, are of the same total area, with the coils of receiving side and the transmitting side both tightly wound with 1500 × 0.01 mm Litz wires, as shown in Fig. 3. The simulation parameters of rectangular coils with different aspect ratios are shown in Table 1.

**Table 1:** Simulation Parameter.

Wire diameter	Core strip thickness	Core width			Distance between coils	Number of turns
5 mm	5 mm	radial core	intersectional shaped core		30 mm	10
		10 mm	50 × 200 cm 13.3 mm	100 × 100 cm 12.1 mm		

The simulation results of the coupling coefficient of each coil in MAXWELL are shown in Table 2. It can be seen from Table 2 that under the same coil and core area, the coupling effect of the rectangular coil with radial core with different aspect ratio is better than that of intersectional shaped core, which has higher coupling coefficient and mutual inductance.

**Table 2:** Simulation result.

Coil size /mm	Core structure	Coupling coefficient
100 × 100	radial core	0.357
	intersectional shaped core	0.346
200 × 50	radial core	0.339
	intersectional shaped core	0.328

**Figure 4:**
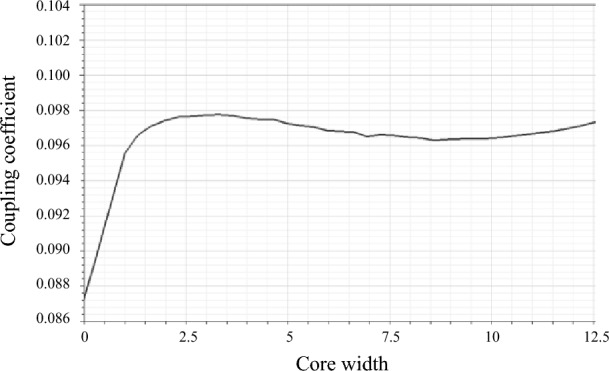
Curve of the coupling coefficient with changing of the width of core.

Meanwhile, to verify the effect of magnetic core thickness on coupling coefficient, the simulation results in MAXWELL showing the curve of the coupling coefficient and change of width of the magnetic strips of the rectangular coil with radial core in Fig. 3c are presented as Fig. 4. The distance between transmitting and receiving coil is 100mm.

It can be seen from Fig. 4 that the coupling coefficient k is related to the magnetic core width. Under the current parameters, the coupling effect of coil with radial core is optimal when the strip width is 2.5 mm. Therefore, the magnetic core width is taken as an optimization parameter in the coupling coefficient optimization.

WPT systems can achieve the maximum output power at the optimal distance, or the coils will work in the over-coupled state, in which the frequency-splitting phenomenon would occur and the output power would decrease^34^. Therefore, the distance between transmitting and receiving coils cannot be chosen as an optimization variable.

### Calculation of coupling coefficient based on magnetic reluctance

**Figure 5:**
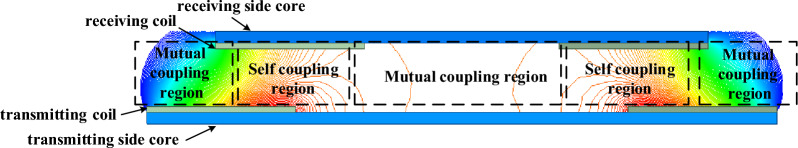
Magnetic field segmentation.

The two-dimensional equivalent models of the cores and coils are shown in Fig. 5. When the magnetic resistance in the core is ignored, the magnetic flux lines can be segmented into the self-coupling region and the mutual coupling region, as shown in Fig. 5.

**Figure 6:**
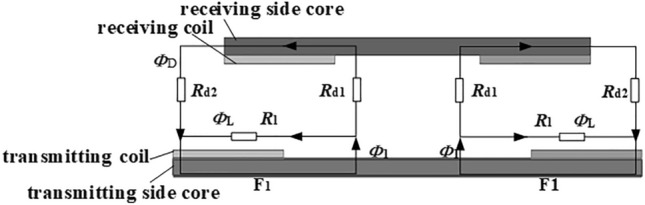
Equivalent magnetic circuit model.

The magnetic field distribution can be analyzed in two-dimensional cross-section magnetic field. Assuming that there is a magnetic resistance *R*_D_ in the magnetic leakage area and a magnetic resistance *R*_L_ in the mutual magnetic area, the equivalent magnetic circuit model can be constructed, as shown in Fig. 6.

The coupling coefficient *k*_1_ can be obtained by (3):

3 $$k_{1} = \frac{{R_{{\text{l}}} }}{{R_{{\text{d}}} + R_{{\text{l}}} }}$$

where *R*_d_ is

4 $$R_{d} = \frac{1}{{\frac{1}{{R_{d1} }} + \frac{1}{{R_{d2} }}}}$$

**Figure 7:**
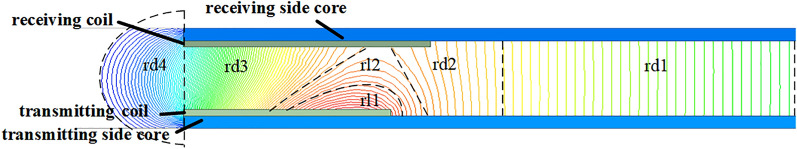
X-direction magnetic flux tubes segmentation.

**Figure 8:**
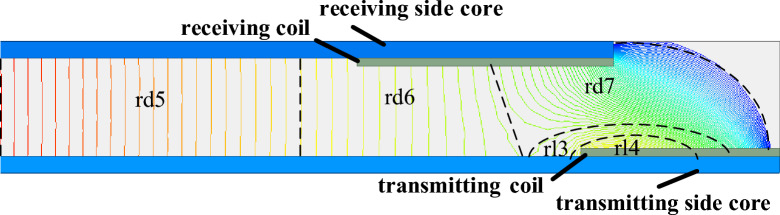
Y-direction magnetic flux tubes segmentation.

With the electromagnetic field simulation results combined, as shown in Fig. 5, the equivalent magnetic circuit can be divided into several magnetic flux tubes^35^. The X-direction magnetic flux tubes are divided as shown in Fig. 7, and the Y-direction magnetic flux tubes are shown in Fig. 8. The magnetic resistance of each flux tube corresponds to the magnetic resistance distribution in Fig. 6.

Usually, the permeability of copper is about the same as that of air, and the permeability of magnetic strip is more than 2000 times that of air. The main magnetic resistance in the magnetic circuit is the magnetic resistance of the air. To simplify the calculation, the magnetic resistance of the magnetic core strips can be ignored. In a single medium, the magnetic resistance is:

5 $$R_{m} = \frac{l}{\mu \cdot A}$$

where *l* is the length of the magnetic circuit, *μ* is the magnetic permeability of the medium, and *A* is the cross-sectional area of the magnetic circuit.

In Fig. 7, the mutual magnetic flux *Φ*_d1_ returns to the core from the central part of the core through the air magnetic resistance *r*_d1_, *r*_d2_, *r*_d3_, and *r*_d4_, and forms a closed loop. The air magnetic resistance, *r*_d1_ can be approximated as a rectangular column flux tube magnetic resistance. *r*_d2_ and *r*_d3_ can be approximated as trapezoidal column flux tube magnetic resistances. And *r*_d4_ can be approximated as a semi-cylindrical flux tube magnetic resistance. The leakage magnetic flux *Φ*_L1_ returns to the magnetic core through the air magnetic resistance *r*_l1_ and *r*_l2_ to form a closed loop. *r*_l1_ can be approximated as a semi-elliptic cylinder flux tube magnetic resistance, and *r*_l2_ can be approximated as an annular fan column flux tube magnetic resistance. In Fig. 8, the magnetic flux *Φ*_d2_ returns to the magnetic core through the air magnetic resistance *r*_d5_, *r*_d6_, and *r*_d7_ to form a closed loop. *r*_d5_ can be approximated as a rectangular column flux tube magnetic resistance, *r*_d6_ is approximated as a trapezoidal column flux tube magnetic resistance, and *r*_d7_ is approximated as an annular fan column flux tube magnetic resistance. Magnetic flux *Φ*_d2_ returns to the core through air magnetic resistance *r*_l3_ and *r*_l4_ to form a closed loop. *r*_l3_ can be approximated as a semi-elliptic cylinder flux tube magnetic resistance, whereas *r*_l4_ can be approximated as an annular fan column flux tube magnetic resistance. The shapes and parameters of flux tubes are shown in Table 3.

**Table 3:** Magnetic flux tubes shapes and parameters.

Magnetic flux tubes shape	Legend
Rectangular Column	
Trapezoidal Column	
Semi-cylinder	
Semi-elliptic Cylinder	
Annular Fan Column	

The calculation of magnetic reluctance of each magnetic flux tube is shown in (6), where, *r*_1_､*r*_2_､*r*_3_､*r*_4_､*r*_5_ are the magnetic reluctance of rectangular column, trapezoidal column, semi-cylinder, semi-elliptic cylinder, annular fan column, respectively.

Based on Fig. 6, the total leakage magnetic resistance and the mutual magnetic resistance in X and Y directions can be calculated, as shown in (7):

6 $$\left\{ {\begin{array}{*{20}c} {r_{1} = \frac{h}{l1 \cdot w}} \\ {r_{2} = \frac{2h}{{\left( {lt1 + lt2} \right) \cdot w}}} \\ {r3 = \frac{1}{0.26 \cdot w}} \\ {r4 = \frac{{\pi \cdot (1.5 \cdot (rt1/2 + rt2/2) - \sqrt {\frac{{(rt1/2) \cdot \left( {rt2/2} \right)}}{8}} )}}{2 \cdot w \cdot (rt1/2 + rt2/2)}} \\ {r5 = \frac{{\pi \cdot \left( {\frac{h}{{sin\left( {a1/2} \right)}} + rt1} \right)}}{4 \cdot rt1 \cdot w}} \\ \end{array} } \right.$$

7 $$\left\{ {\begin{array}{*{20}c} {R_{d1} = \frac{1}{{\frac{1}{{r_{d1} + r_{d3} }} + \frac{1}{{r_{d2} + r_{d4} }}}}} \\ {R_{{{\text{l}}1}} = \frac{1}{{\frac{1}{{r_{{{\text{l}}1}} }} + \frac{1}{{r_{{{\text{l2}}}} }}}}} \\ {R_{d2} = \frac{1}{{\frac{1}{rd5} + \frac{1}{rd6}}}{ + }rd7} \\ {R_{l2} = \frac{1}{{\frac{1}{rl3} + \frac{1}{rl4}}}} \\ \end{array} } \right.$$

where *R*_d1_ and *R*_d2_ are mutual magnetic resistances in X and Y directions, respectively, and *R*_l1_ and *R*_l2_ are leakage magnetic resistances in X and Y directions, respectively.

Since the transmitting and receiving magnetic core strips in the diagonal direction are not in the same plane, the flux tubes cannot be divided by Maxwell 2D. If the receiving side core and coil were rotated angle *α*, the diagonal magnetic strips of transmitter and receiver would be located in the same plane. And the flux tube distribution is totally the same as that in the X direction. So that, in the diagonal direction, the total leakage magnetic resistance and mutual magnetic resistance *R*_d3_ and *R*_l3_ can be calculated by changing the corresponding parameters of models.

Since *R*_d3_ is the total mutual magnetic resistance obtained after the torsion angle *α*, there must be an error, which can be reduced by introducing the correction parameter *β* related to *α*. The formula of the total magnetic resistance is:

8 $$R_{d} = \frac{1}{{\frac{1}{{R_{d1} }} + \frac{\beta }{{R_{d3} }} + \frac{1}{{R_{d2} }}}}$$

The parameters of magnetic flux tubes can be measured through Maxwell 2D cross-sectional simulation results of models of different transmitting and receiving coils and cores, where the parameter items are the same as shown in Table 3. The measurement results are fitted and calculated based on the least square method to obtain the parameter distribution of flux tubes under different design parameters, as shown in (9).

9 $$\left\{ {\begin{array}{*{20}c} {rt1x = 0.631 \cdot x1 - 0.03805 \cdot d1^{2} + 1.569 \cdot d1 - 1.456} \\ {rt1y = - 0.03805*d1^{2} + 1.569*d1 + 8.168} \\ {rt2 = 0.146*h + 0.0126} \\ {R = h} \\ {t1 = 0.5 \cdot l2 - yt2x - l1x} \\ {t2 = 0.0119 \cdot x2^{2} - 1.0571 \cdot x2 + 5.538} \\ {ax = \pi } \\ {ay = \pi } \\ {a3 = \arctan (\frac{h}{0.5 \cdot l1 - 0.5 \cdot x1})} \\ {l1x = 0.286 \cdot x1 + 0.312 \cdot l{2} - 4.59} \\ {l{\text{1y = - 0}}{.1943 } \cdot {\text{x2 + 8}}{.171}} \\ {yt1x = {0}{\text{.5}} \cdot l{2} - {0}{\text{.286*}}x1 - {7}{\text{.41;}}} \\ {yt2x = 0.03471 \cdot d1^{2} - 1.486 \cdot d1 + yt1x + \frac{l2 \cdot (l1 - l2)}{{1.25 \cdot l1}};} \\ {lt1y1 = 0.5536 \cdot x2 + 6.607 + 0.5 \cdot rt1y} \\ {lt2y1 = 0.7214 \cdot x2 - 2.457} \\ {lt1y2 = {0}{\text{.5}} \cdot l{2} - lt1y1 - l{\text{1y}}} \\ {lt2y2 = lt1y2 - 0.5 \cdot t} \\ \end{array} } \right.$$

where *l*1 is the length of transmitter coil, *l*2 is the length of receiver coil, *d*1 is the width of the winding of transmitter coil, *d*2 is the width of the winding of receiver coil. × 1, × 2 are as shown in (10), respectively. The representations of the symbols of flux tubes are shown in Table 4.

10 $$\left\{ {\begin{array}{*{20}c} {x1{ = }\frac{l1 - l2}{2}} \\ {x2{ = }\frac{d1 - d2}{2}} \\ \end{array} } \right.$$

**Table 4:** Magnetic flux tubes shapes and parameters.

Symbol	Item	Magnetic flux tube	Direction
*rt*1*x*	lengths of the long axis	Semi-elliptical cylinder	X
*rt*1*y*			Y
*r*t*2*	length of the short axis		X, Y, diagonal
*R*	Radius	Semi-cylindrical cylinder	
*l*1*x*	Lengths	Rectangular columns	X
*l*1*y*			Y
*ax*	Angles	Circular fan columns	X
*ay*			Y
*ad*			diagonal
*t*1	Widths		X
*t*2			Y
*yt*1*x*	Upper base widths	Trapezoidal column	X
*yt*2*x*	Lower base widths		
*lt*1*y*1	Upper base widths	Left trapezoidal column	Y
*lt*2*y*1	Lower base widths		
*lt*1*y*2	Upper base widths	Right trapezoidal column	
*lt*2*y*2	Lower base widths		

**Figure 9:**
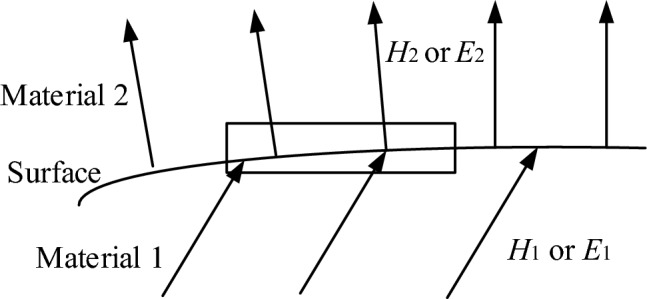
Tangent directions on surface of different materials in electromagnetic field.

**Figure 10:**
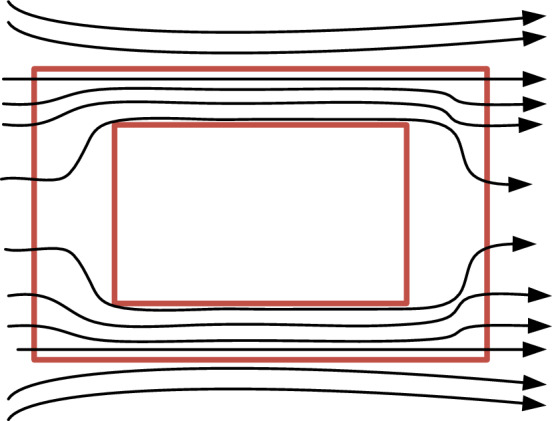
Distribution of magnetic lines near the core.

According to the boundary conditions on the surfaces of different materials in the electromagnetic field, the tangent angle ratio of the magnetic lines on both sides of the surface is the same as the ratio of the magnetic permeability^36^, as shown in Fig. 9. The magnetic permeability of copper is approximately equal to that of air, much smaller than that of the core material. The magnetic field lines on the surface of the magnetic core can be approximately considered to be perpendicular to the surface before entering the magnetic core from the air, and become parallel after entering the core. And the magnetic induction intensity in the core is much greater than that in the air far from the magnetic core. Therefore, the magnetic lines near the core can be considered as being distributed near around the magnetic core area, as shown in Fig. 10.

With the magnetic core reluctance being ignored, the equivalent path of the air reluctance can be regarded as mainly concentrated near the magnetic core^37^.

When there is a displacement of the receiving coil in the X direction, it can be considered that the relative displacement between the two coils leads to the increase of the cross-sectional area of the equivalent magnetic circuit. And the correction variable *o* of core width parameter *w* in (6), as shown in (11):

11 $$o \approx \frac{{(0.5l1 - \Delta x)^{2} }}{0.5l1*0.5m1}$$

The coupling coefficient *k*_2_ after the movement can be obtained by (12):

12 $$k_{2} = \frac{{R_{{\text{l}}} ^{\prime}}}{{R_{{\text{d}}} ^{\prime} + R_{{\text{l}}} ^{\prime}}}$$

**Figure 11:**
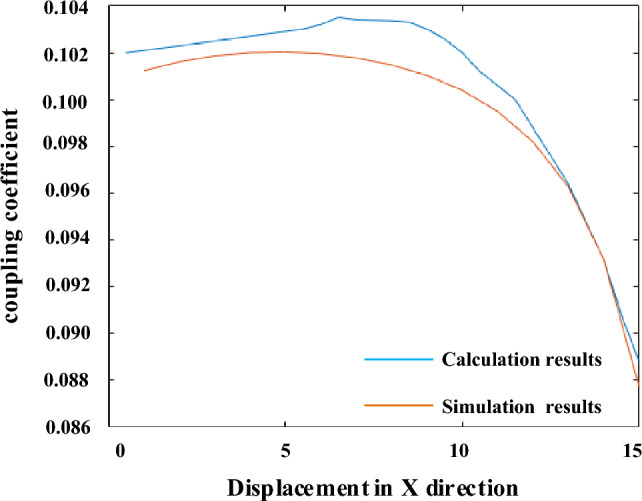
Simulation and calculation results.

The length and width of transmitting and receiving coils are selected as 200 and 100 mm respectively, the core width is 20 mm, and the spacing is 50 mm. The comparison between the simulation and calculation results when the receiving coil is displaced is shown in Fig. 11.

It can be seen from Fig. 11 that the error between the simulation and calculation results is less than 5%. Therefore, the reliability of the calculation model is credible.

## Coil optimization based on NSGA-II algorithm

### Optimization model

The length, width and turns of the transmitting and receiving coils are the main variables of the coil design. It can be seen from the above that the core width is also an important factor affecting the coil coupling effect. Therefore, this paper takes the length and the turns of the transmitting and receiving coils, and the width of magnetic strips of the cores as the optimization variables of the optimization model of the dynamic wireless charging coil. Considering the practical requirements of the dynamic wireless charging system, the width of coils, the distance between transmitting and receiving coils and the diameter of Litz wire are given as 730 mm, 350 mm and 5.5 mm respectively. Meanwhile, the width of the transmitting and receiving side cores, and the length of transmitting and receiving coils are set to be the same respectively.

Referring to (2), in order to improve the efficiency of the wireless charging system, a higher coupling coefficient *k*_1_ is desired within the constraint range. NSGA-II is an algorithm for solving the minimum value of the objective function. Therefore, in order to improve the coupling coefficient *k*_1_, the actual optimization function required should be changed to:

13 $$\it \it {\text{f}}_{{1}} {\text{ = 1 - k}}_{{1}} \begin{array}{*{20}c} {} & {} \\ \end{array}$$

where *k*_1_ is the coupling coefficient when there is no dis-alignment between the center of the transmitting and receiving coil, which can be obtained from (3).

The process of moving receiving coil 10 cm is a part of displacement in EV dynamic process. In order to ease the efficiency reduction caused by the receiving coil moving away from the current transmitting coil with the vehicle moving forward in actual work, the sensitivity of the coupling coefficient with the vehicle displacement in the forward direction *Δk* in the moving direction of the receiving side coil in the vehicle should be optimized, which is the rate of change of the coupling coefficient after the vehicle moves forward per unit length. It can ensure that the high-power output range of each transmitting side coil is longer. In this paper, the length is chosen as 100 mm. It can be taken as another optimization objective *f*_2_.

14 $$f_{2} = \Delta k = (k_{1} - k_{2} )/k_{1}$$

where *k*_2_ is the coupling coefficient after the receiving edge is displaced by unit length along the X direction, which can be obtained from (12).

Therefore, the optimization objective function is shown in (15):

15 $$\left\{ {\begin{array}{*{20}c} {{\text{min}}\begin{array}{*{20}c} {} & {{\text{f}}_{{1}} {\text{(x}}_{1} , x_{2} ,x_{3} ,x_{4} ,x_{5} )} \\ \end{array} } \\ {{\text{min}}\begin{array}{*{20}c} {} & {{\text{f}}_{{2}} {\text{(x}}_{1} , x_{2} ,x_{3} ,x_{4} ,x_{5} )} \\ \end{array} } \\ \end{array} } \right.\begin{array}{*{20}c} {} & {} \\ \end{array}$$

where *x*_1_, *x*_2_ is the length of transmitting and receiving coils respectively, *x*_3_, *x*_4_ is the number of turns of transmitting and receiving coils respectively, and *x*_5_ is the width of strips of the core.

Considering the practical requirements of a wireless charging system, the constraints on the coil length, width, turns and core width are

16 $${\text{x}}_{i} \_min \le x_{i} \le x_{i} \_max\left( {{\text{i}} = 1,2,3,4,5} \right)$$

17 $$\left\{ {\begin{array}{*{20}c} {x_{3} \cdot 2p \le x_{1} } \\ {x_{4} \cdot 2p \le x_{2} } \\ {x_{3} \cdot 2p \le w_{{\text{d}}} } \\ {x_{4} \cdot 2p \le w_{{\text{d}}} } \\ \end{array} } \right.$$

where *p* is the diameter of Litz wire, which is given as 5.5 mm according to the working current 20A, and *w*_d_ is the width of transmitting and receiving coils, which is given as 730 mm according to the width of common automobile chassis.

### Optimization based on NSGA-II algorithm

Multi-objective optimization problem is usually solved by multi-objective optimization algorithm. NSGA algorithm is improved based on Simple Genetic Algorithm (SGA), and NSGA-II algorithm is an improved version of NSGA^38^, which adopts fast non dominated sorting strategy and elite strategy on NSGA algorithm. The congestion function is used to replace the sharing function, which greatly reduces the computing time and complexity^39^.

**Figure 12:**
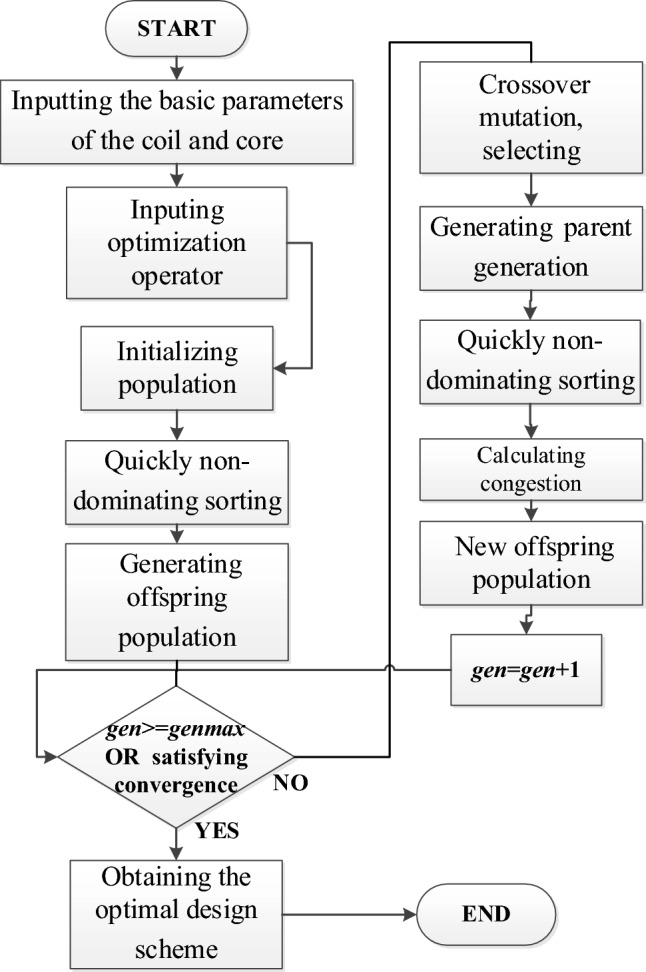
Coil optimization design process using NSGA-II algorithm.

NSGA-II algorithm is an algorithm to obtain the minimum solution of each optimization objective function through continuous screening of best individuals in the offspring. The key steps are fast non-dominated sorting and crowding distance sorting. Specifically, the specific process of solving the coil optimization design problem is shown in Fig. 12.

**Figure 13:**
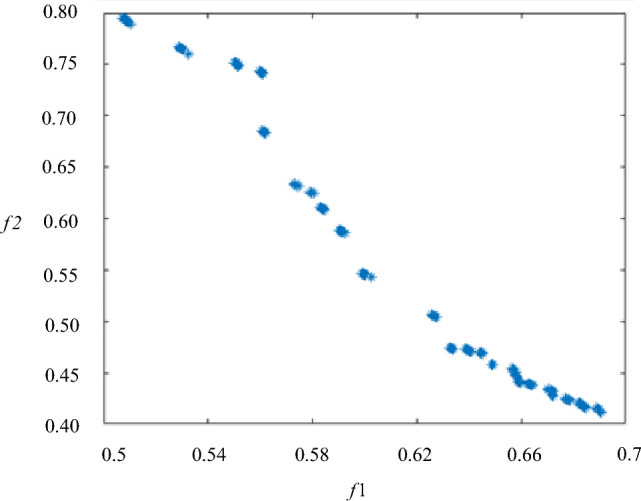
Optimization scheme.

In the actual solution process, when the maximum algebras *genmax* selected are 400, 500, 600 generations respectively, and the distribution of solution sets obtained by optimization is basically the same. It indicates that the function has converged in 400 generations in MATLAB 2020B, as shown in Fig. 13. There is no element simulation model was built in the process after the optimization model constructed. Compared with the trial-and-error method and the MAXWELL/MATLAB joint simulation optimization method, it can save lots time of simulation operations.

It can be seen from Fig. 13 that the variation trends of the two optimization objectives are opposite. When the 1-*k* is larger, *Δk* is smaller. And the smaller 1-*k* is, the larger *Δk* gets. Considering the higher importance of k in the DWPT system, the weight 0.7:0.3 is taken as an example. The 910 mm scheme is chosen as the optimization design scheme. When the weight ratios are 1:0 and 0:1, the solution sets are 850 mm scheme and 760 mm scheme, respectively. The schemes are as shown in Table 5, where *T* is the transmitting side and *R* is the receiving side.

**Table 5:** Optimization Schemes.

Scheme	Side	Length of coil /mm	Number of turns	Width of core /mm	*k*_1_	Δ*k*
910	T	910	29	85	0.442	6.8%
	R	470	25			
760	T	760	14	90	0.480	7.9%
	R	480	32			
850	T	850	34	110	0.321	4.1%
	R	400	35			

### Experiment and verification

**Figure 14:**
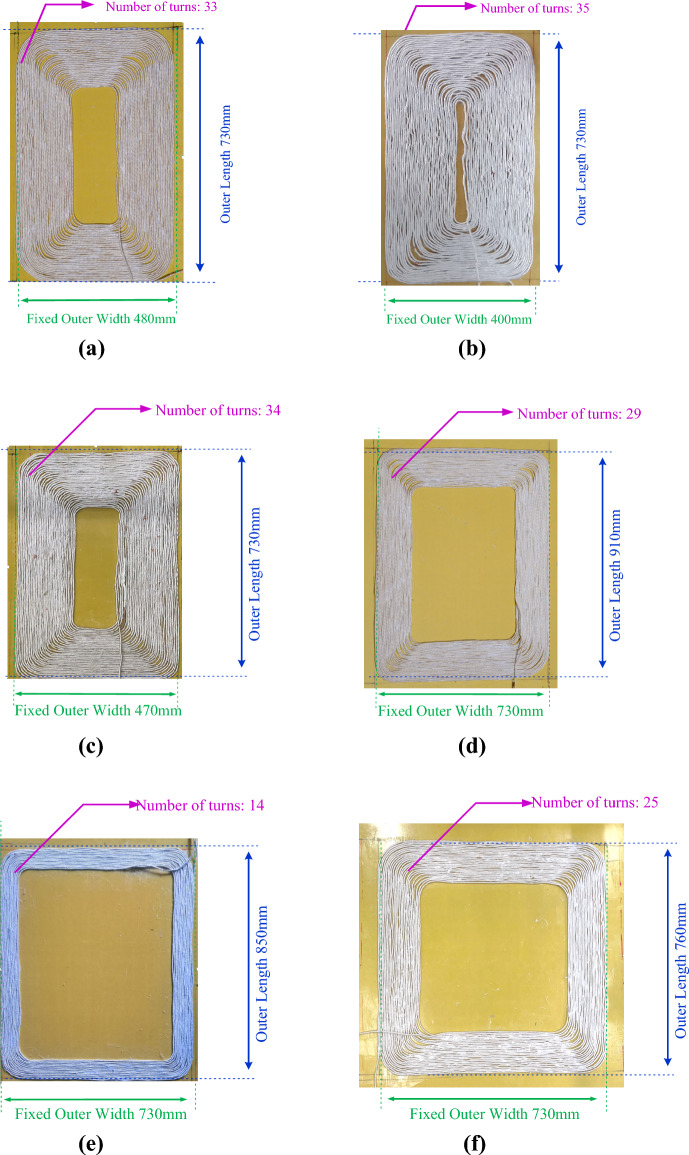
The coils and the cores. (**a**) Receiving coil of 910mm scheme. (**b**) Receiving coil of 850mm scheme. (**c**) Receiving coil of 760mm scheme. (**d**) Transmitting coil of 910mm scheme. (**e**) Transmitting coil of 850mm scheme. (**f**) Transmitting coil of 760mm scheme.

In order to verify the optimization results, the finite element simulation is conducted according to the parameters of the optimization schemes. The corresponding resonant coil is wound and the radial magnetic core is made, as shown in Fig. 14.

In the simulation and experiment, the center of the transmitting and receiving radial magnetic core and coil with different parameters was aligned.

**Figure 15:**
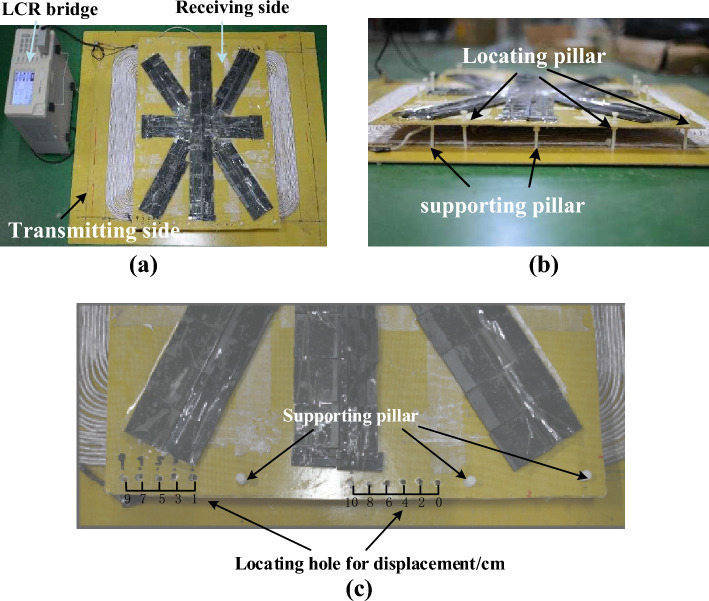
The experimental platform. (**a**) Experimental platform top-view. (**b**) Experimental platform side-view. (**c**) Location hole and support pillar.

The experimental platform is shown in Fig. 15.

As shown in Fig. 15, the LCR bridge measures the self-inductance and mutual inductance of the transmitting and receiving coils under different displacements. The coupling coefficient is calculated by (18).

18 $$k = \frac{M}{{\sqrt {L_{1} L_{2} } }}$$

The positions of location holes coincide with the locating pillars under the corresponding displacement. The supporting pillars is located at the edge of the receiving coil to support the receiving coil and magnetic core.

**Figure 16:**
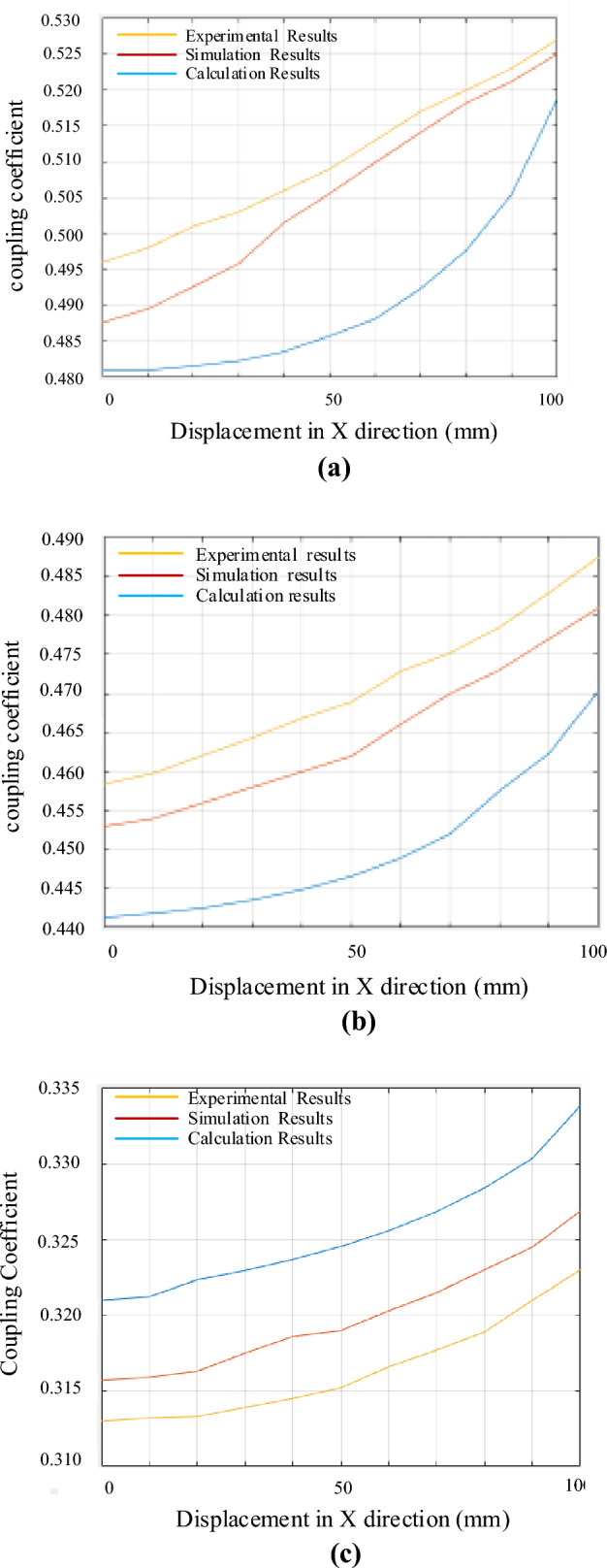
Curve of the coupling coefficient with the X distance displacement. (**a**)760 mm scheme. (**b**) 910 mm scheme. (**c**) 850 mm scheme.

The curve of the coupling coefficient with the X-direction displacement under different parameters can be obtained, as shown in Fig. 16.

It can be seen from Fig. 16 that the experimental coupling coefficients corresponding to different parameters are 0.496, 0.458, 0.313 respectively. Under 100 mm displacement, the sensitivities of coupling coefficient are 0.527, 0.487, 0.334 respectively. In terms of coupling coefficient and sensitivity, under different parameters, the error between simulation results and experimental data is less than 5% on average. The curves of coupling coefficient of different coils with X distance displacement calculated from the experimental results are basically the same as the simulation results and the calculation results based on magnetic circuit analysis. Compared with those of 850 mm scheme, in the 910 mm scheme calculation results, *k*_1_ increases by 27.3%, and *Δk* decreases by 39.7%. Meanwhile, compared with those of 760 mm, in the 910 mm scheme calculation results, the 910 mm scheme *k*_1_ decreases by 8.6%, and *Δk* increases by 16.2%. It proves that the optimization is effective. In summary, the multi-objective optimization results based on magnetic circuit analysis and analytical calculation are accurate. Since the displacement distance is short ,the trend of curve is still in a brief upward phase as shown in Fig. 11.

**Figure 17:**
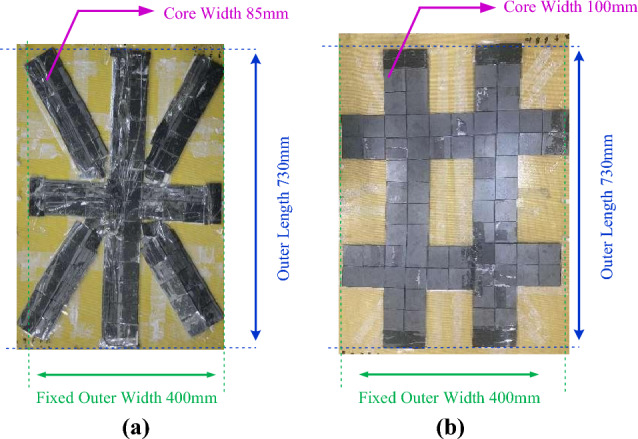
The radial and intersectional shaped receiving cores. (**a**) Radial core. (**b**) Intersectional shaped core.

In order to verify the advantages of radial magnetic core, the transmitting and receiving side of the intersectional shaped magnetic cores of the 910 mm scheme was made for comparative experiments, which keep the coil the same, and the total area of the magnetic core equal. For instance, the receiving side are shown in Fig. 17. The core strips are composed of 5*5*0.2cm and 5*0.5*0.2mm magnetic plates. The Diagonal direction strips near centre are composed of triangular magnetic plates and 5*0.5*0.2mm rectangular magnetic plates to fit as closely as possible.

The results of coupling coefficient comparison under different magnetic cores are shown in Table 6. The experimental results show that the radial magnetic core does have a higher coupling coefficient.

**Table 6:** Experimental results.

Optimization scheme	Displacement /mm	Core structure	Coupling coefficient
910 mm	0	radial core	0.458
		intersectional shaped core	0.425
	10	radial core	0.487
		intersectional shaped core	0.453

## Conclusion

A new radial magnetic core for rectangular coils is adopt in this paper. It is verified that radial magnetic core has a higher coupling coefficient compared with the common magnetic core in the same area through simulation and experimental results. Based on the magnetic circuit analysis, the analytical expressions of the coupling coefficient of the new cores and coils are obtained. The multi-objective optimization solutions are carried out by NSGA-II algorithm, and the coil optimization design schemes are obtained. When the width of the transmitting coil is restrained to 730 mm, in the selected 910 mm optimal scheme, compared with those in 850 mm scheme, *k*_1_ increases by 27.3%, and *Δk* decreases by 39.7%. Compared with those in 760 mm scheme, *k*_1_ decreases by 8.6% and *Δk* increased by 16.2%. Therefore, it proves that the optimization is effective. The function is converged in 400 generations, and there is no finite element simulation model established during optimization, which shortens the optimization process. The optimization results show that the optimization method discussed in this paper, which is combined of magnetic field segmentation method and parameter fitting, can reduce the complexity of optimization process and optimization calculation time. Through simulation and experiments, it is verified that the analytical method is relatively accurate.

### Supplementary Information

Supplementary Table 1.

## Data Availability

The authors confirm that the data supporting the findings of this study are available within the article or its supplementary materials and the legend for figures has been added in the Supplementary file.
